# ABA activated SnRK2 kinases: an emerging role in plant growth and physiology

**DOI:** 10.1080/15592324.2022.2071024

**Published:** 2022-05-04

**Authors:** Md.Mahadi Hasan, Xu-Dong Liu, Muhammed Waseem, Yao Guang-Qian, Nadiyah M. Alabdallah, Mohammad Shah Jahan, Xiang-Wen Fang

**Affiliations:** a State Key Laboratory of Grassland Agro- College of Ecology, Lanzhou University, Lanzhou 730000, Gansu Province, China; b Department of Biology, College of Science, Imam Abdulrahman Bin Faisal University, Dammam 31441, Saudi Arabia; c Department of Horticulture, Sher-e-Bangla Agricultural University, Dhaka, Bangladesh

**Keywords:** Abiotic stress, land plants, phytohormone, protein kinases, phosphorylation, stomatal closure

## Abstract

Members of the SNF1-related protein kinase 2 (SnRK2) family are plant-specific serine or threonine kinases that play a pivotal role in the response of plants to abiotic stresses. Members of this plant-specific kinase family have included a critical regulator (SnRK2) of abscisic acid (ABA) response in plants. Plant organ development is governed substantially by the interaction of the SnRK2 and the phytohormone abscisic acid (ABA). Recent research has revealed a synergistic link between SnRK2 and ABA signaling in a plant’s response to stress such as drought and shoot growth. SnRK2 kinases play a dual role in the control of SnRK1 and the development of a plant. The dual role of SnRK2 kinases promotes plant growth under optimal conditions and in the absence of ABA while inhibiting the growth of plants in response to ABA. In this review, we have uncovered the roles of ABA-activated SnRK2 kinases in plants, as well as their physiological mechanisms.

## Introduction

**Figure 1.**
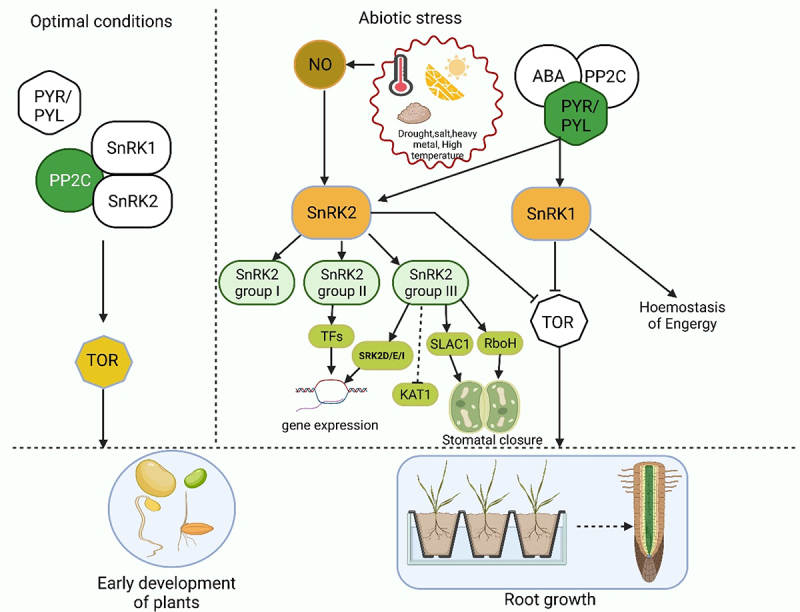
The simple pathway SnRK2s pathways involved in plant response to abiotic stress and SnRK2 kinases perform dual functions in plants adapted from Belda-Palazón et al. (2020). SnRK2s boost growth under optimal conditions. SnRK2s are involved in the formation of SnRK1 repressor complexes which also contain PP2Cs in the lack of ABA. SnRK1 sequestration in these complexes is critical for preventing SnRK1 from interacting with TOR and thus allowing growth while conditions are suitable. SnRK2s prevent the growth in response to stress. SnRK2 and PP2C-containing SnRK1 repressor complexes dismantle in the involvement of ABA via classical ABA signaling, which involves the sequestration of PP2Cs by ABA-bound PYR/PYL receptors.SnRK2s and SnRK1 are released when the complexes are disassembled, triggering stress responses and inhibiting growth. This is achieved in part through direct TOR suppression by SnRK1, but it is also possible that SnRK2 kinases are involved in the process.Inactive components are spotlighted in white, whereas active components are displayed in other colors.

Throughout their life cycle, plants are constantly bombarded with harmful environmental factors like abiotic (drought, salt, heavy metal etc.) and biotic stressors (insects, pathogens etc.).^1–4^ Plants must be capable of sensing environmental parameters and must be able to respond to these changes by employing a variety of defense mechanisms to ensure their survival.^5–7^ A variety of specific signaling pathways, including protein kinases and phosphatases, are involved in recognizing stress signals and transmitting these signals to various cellular compartments. In plants, SNF1-related kinases (SnRKs) are ubiquitous to all eukaryotic species. Previous studies have demonstrated that SnRK2 kinases are critically involved in the response of plants to environmental stress such as drought.^8,9^ When plants are stressed, they accumulate more ABA that causes defensive stress responses through ABA-dependent or ABA-independent pathways, both of which activate several SnRK2s.^10^ The direct phosphorylation of numerous downstream targets, such as SLAC1, KAT1, AtRbohF, and transcription factors necessary for the stress responsive gene expression, regulates the plant response to ABA through SnRK2s pathways. As a result, these downstream targets make defensive stress responses easier (Figure 1).^11,12^

The SnRK2 subfamily includes ABA-dependent kinases and can be found in the whole plant. They are essential components for the response of plants to ABA, both in optimal and adverse environmental conditions, during plant growth, flowering time, seed maturity, and germination.^13^ ABSCISIC ACID INSENSITIVE5 (ABI5) is a transcription factor that is considered to be a major regulator of abscisic acid (ABA) mediated seed germination and plant growth. Phosphorylation of ABI5 by SnRK2 had a direct influence on the floral transition.^14^ ABI5 seems to be the only known regulator of floral initiation in plants via the ABA signaling transduction pathway.^15^ Hwang et al. (2019)^16^ demonstrated that the other bZIP-type transcription factors, ABF3 and ABF4, promote flowering by increasing *SUPPRESSOR OF OVEREXPRESSION OF CONSTANS1 (SOC1)* expression in response to drought, while the tomato OST1 kinase enhances flowering by phosphorylating the NAC-type transcription factor VOZ1 in under drought stress conditions.^17^ *FLOWERING LOCUS C (FLC)* transcription is activated by the transcription factor *ABSCISIC ACID INSENSITIVE MUTANT 5 (ABI5)*, which binds directly to its promoter. Thus, the ABI5-FLC module negatively regulates flowering time in *Arabidopsis* and the ABA-activated SnRK2.6 (OST1) is essential for this regulation.^18^ Inhibition of seed germination and seedling growth are caused by the activation of three SnRK2 family members (SnRK2.2, SnRK2.3, and SnRK2.6/OST1), which phosphorylates a large number of downstream effectors in response to ABA.^19–21^ SnRK2.2 and SnRK2.3 are expressed predominantly in seeds, which have similar functions in ABA suppression of seed germination.^22^ In this review, we concentrate on the regulation of ABA-activated SnRK2 kinases in plant growth and physiology. We pay special attention to the dual role of SnRK2 kinases in the control of SnRK1 and plant development, as well as exploring their underlying mechanisms. Finally, we give an overview of SnRK2 protein kinases in plants and how they assist plants in adapting to a dynamic environment.

## SnRK2 kinases in the earliest land plants

The phytohormone ABA accumulates in response to stress conditions, causing stomatal closure and storage of soluble carbohydrates, which helps preserve cellular activities from being dehydrated.^23^ SnRK2 kinases phosphorylate proteins in response to ABA signaling.^24,25^ In angiosperms, SnRK2s are a key component of ABA signaling pathways mediated by ABA receptors (PYR/PYL/RCAR) and protein phosphatase 2Cs (PP2CAs), and are also divided into three subclasses: I, II, III based on amino acid sequence similarity and ABA response.^26,27^ However only D-rich SnRK2s (subclass II and III) are activated by ABA, osmotic stress activates all SnRK2 members except *Arabidopsis* SRK2J/SnRK2.9 (subclass I). As a result, SnRK2s from subclass II and III are categorized as ABA-responsive SnRK2s, whereas SnRK2s from subclass I are classified as ABA-unresponsive SnRK2s. SnRK2s of subclass I are documented in a number of angiosperms but not in bryophytes or algae. In Arabidopsis, SnRK2 subclass II (SRK2C/SnRK2.8 and SRK2F/SnRK2.7) have been identified, which are both activated in response to osmotic stress but very slightly in response to ABA.^28^ This class includes the first reported SnRK2, PROTEIN KINASE ABA 1 (PKABA1) from wheat.^29^ Subclass II-type SnRK2 has also been found in lycophytes (*Selaginella tamariscina*), suggesting that it might be an intermediary molecule between subclass III SnRK2 in algae and subclass I SnRK2 in seed plants.^30^ Subclass III-type SnRK2s (SnRK2.2, SnRK2.3, and SnRK2.6/OST1) serve as a focal point for ABA signaling.^25^ SnRK2.2 and SnRK2.3 play a part in the suppression of plant growth and yield regulated by ABA.^31,32^ Phosphorylation of numerous regulatory proteins, like ion channels (SLOW ANION CHANNEL-ASSOCIATED 1, SLAC1 and potassium channel protein, KAT1), and modulation of stress-responsive genes, is more likely through the activation of *ABSCISIC ACID RESPONSIVE ELEMENT BINDING FACTOR (ABF)* to regulate the response to ABA via SnRK2s.^13,31^ Thus, *Arabidopsis* PP2CAs inhibit subclass III SnRK2 activities via direct contact, which negatively regulates ABA signaling. When ABA binds to PYR/PYL/RCAR, that particularly sequester the PP2CAs, the inhibition is abolished, allowing SnRK2 to be activated.^33,34^ SnRK2s are conserved evolutionarily and hereditarily across land plants. *Physcomitrella patens* is a well-known model moss with a genome containing four SnRK2 genes (PpSnRK2A/2B/2C/2D) categorized into subclass-III.^35^ PpSnRK2A/PpOST1 recovers the ABA responsiveness of stomatal closure in the *Arabidopsis snrk2.6/ost1* mutant, whereas loss of PpSnRK2A/PpOST1 causes in defective ABA-responsive stomatal closure in moss.^36^ Furthermore, it is unknown where the Ppsnrk2a/Ppost1 plant lacks ABA sensitivity in the moss protonemata, whereas ABA responses as well as resistance to dehydration and osmotic stress are well reported.^37^

In recent years, It was elucidated that an ancestral subclass III SnRK2-based signaling unit containing ABA and an upstream Raf-like kinase (ARK) defends the moss *Physcomitrella patens* entirely from drought.^38^ Subclass III SnRK2s from *Arabidopsis* and the semiterrestrial alga *Klebsormidium nitens* also include all ABA signaling elements apart from ABA receptors, complementing *Physcomitrella snrk2* mutants, while *Arabidopsis* subclass I SnRK2s do not complement *Physcomitrella snrk2* mutants.^39^ The ABA/ARK/subclass III SnRK2 signaling module was established by employing ABA to control a preexisting dehydration response in ancient terrestrial plants. A novel subclass I SnRK2 system was developed in angiosperms that conferred osmotic stress safeguards autonomously within the archaic system.^36^

## Physiological regulation of SnRK2s in stomatal development

Many studies have demonstrated that SnRK2s are effective at controlling abiotic stress responses in plant cells.^40^ ABA triggers a protein kinase called ABA-activated protein kinase (AAPK) in fava bean guard cells during water deficit, and it is implicated in the control of stomatal movement in the plant.^4^ In *Arabidopsis*, the SRK2E/OST1/SnRK2.6 protein kinase is the nearest homolog to the AAPK. Until now, this kinase has been the most extensively investigated in terms of its role in the response of plants to environmental stressors. The wilting phenotype of the *srk2e*/*ost1*/*snrk2.6* mutant occurs because of its inability to deal with a substantial reduction in humidity.^41^ The lack of the kinase activity results in a phenotype in which ABA is not required for stomatal closure. It has been demonstrated that the ABA-induced control of the stomatal aperture is mediated by the OST1 protein,^4^ and it functions upstream of the generation of reactive oxygen species (ROS).^42^ In guard cells, ROS are required to mediate ABA signaling. These substances are required to modulate calcium ion (Ca^2+^) influx through the stimulation of Ca^2+^ channels.^43^

Ca^2+^ influx is widely known as a requirement for stomatal closure. In *Arabidopsis*, the ROS-dependent ABA signaling pathway is mediated by two identical guard cell NADPH oxidases (AtRBOHD and AtRBOHF).^43^ As a result, it is quite possible that OST1 regulates ROS generation via phosphorylation of NADPH oxidases in the cellular membrane. It was revealed that phosphorylation and Ca^2+^ binding are required to stimulate AtRBOHD.^44^ Sirichandra et al. (2009)^45^ demonstrated that OST1 phosphorylates the N-terminal region of recombinant AtRBOHF generated in an *in vitro* bacterial system. OST1 binds with AtRBOHF in a bimolecular fluorescence complementation (BIFC) test, indicating that NADPH oxidase may be a substrate for SnRK2.6/OST1 *in vivo* as previously reported. Currently, there is no direct evidence demonstrating the phosphorylation of *Arabidopsis* NADPH oxidases (AtRBOHS) or the control of AtRBOHS activity in plants. ABA-dependent stomatal closing is mediated through distinct ion channels in guard cells that are designed to respond to the hormone ABA. Guard cell slow-anion channel 1 (SLAC1), which plays a role in response to elevated carbon dioxide (CO_2_) and ABA levels, has recently been found and described on a molecular level.^46,47^ Evidence suggests that SLAC1 is a primary platform of guard cells, which is essential for stomatal movement. Previous studies have demonstrated that the function of this channel is controlled by reversible phosphorylation. The coupling of calcium dependent kinase 21 (CDK21) and calcium-dependent protein kinase 23 (CDPK23) could be essential for the upregulation of the SLAC1.^48^ The inward-rectifying potassium channel 1 (KAT1) is also an ion channel found in *Arabidopsis* guard cells responsible for opening stomata pores.^49^

Additionally, the function of this channel is controlled by phosphorylation, which is mediated by SnRK2.6/OST1 and, by our postulation, the CDPK protein.^50^ However, in the instance of KAT1, phosphorylation has a deleterious impact on the protein’s activity. Contrary to SLAC1, it must be noted that the reduction of KAT1 function is required for stomatal closure. It has been shown in the previous research that SnRK2.6/OST1 phosphorylates both anion (SLAC1) and cation (KAT1) channels that are important for stomatal movements and that these phosphorylations are essential for the closure of stomata caused by water deficiency in the presence of ABA. It has been demonstrated that OST1 phosphorylates and activates SLAC1 in reaction to ABA and in response to an increase in intracellular CO_2_^51^ or ozone (O_3_).^7,52^ Although SnRK2.6/OST1 controls stomatal movement, their control over a plant response to drought is not limited to stomatal movement. Ultimately, SnRK2 plays a vital function in the movement of stomata in plants, and this involvement cannot be ignored.

## Emerging dual role of SnRK2 kinases in plants

Under optimal growth conditions, the TARGET OF RAPAMYCIN (TOR) kinase phosphorylates PYL receptors and disrupts the pathways that suppress PP2Cs, resulting in a twofold inhibition of ABA signaling activation.^53^ Activated SnRK2s phosphorylate and stimulate downstream targets such as (ABF) transcriptional regulators and SLAC1 41. Belda-Palazón et al. (2020)^53^ reported two distinct roles of SnRK2 in plants. SnRK2s boost growth in a lack of ABA: SnRK2s, along with PP2Cs, are necessary to form ‘repressor complexes’ that encapsulate SnRK1.^54^ Sequestration of SnRK1α1 in these complexes is required for root development (mostly in the case of SnRK2.2 and SnRK2.3), and it may account for other documented unexpected impacts of SnRK2 kinases, particularly the impact of SnRK2.6, in improving the growth and metabolism in optimal conditions.^55,56^ The disintegration of these complexes to ABA receptors, that sequesters the PP2C repressors and allows them to be released, is necessitated by the binding of ABA. Several lines of evidence support this. As with SnRK2s, the activation of SnRK1 by ABA necessitates the removal of repression caused by PP2C phosphatases.^56^

ABA diminishes the interaction of SnRK1 with SnRK2 and PP2CA and the interaction between SnRK1 and PP2CA. In the absence of PP2Cs, SnRK1 and SnRK2 are not able to interact with one another. SnRK2s (SnRK2.2/SnRK2.3/SnRK2.6) are essential for suppressing TOR in reaction to ABA,^53^ even though SnRK2s might be indirectly engaged in TOR suppression in the absence of ABA. Nevertheless, in the presence of ABA, SnRK2s suppress growth, which is partially achieved by SnRK1 stimulation taking place more readily under abiotic stress conditions. Plants were capable of regulating development in response to the water supply when the ABA-PP2C-SnRK2 module was bound to the evolutionarily conserved SnRK1–TOR axis, which was previously unattainable. This dual control of SnRK1 by SnRK2 kinases combines growth regulation with environmental factors of the terrestrial ecosystem.

## Conclusions and future perspective

Early studies have shown that SnRK2 kinases play an essential role in plants’ growth and maintenance of flowers. SnRK2-type protein kinases are induced in both ABA-dependent and ABA-independent ways under abiotic stresses, and they play critical roles in the development of stress responses. The interplay of the SnRK2 activation pathway with the ABA-dependent and ABA-independent pathways will be a major direction for future research. Although, the physiological function of ABA-independent SnRK2s is poorly understood, therefore we anticipate that this kinase group will be explored and observed extensively in the future. Future research will answer questions about SnRK2s function and the mechanisms that regulate their activity. Combined “omic” techniques should be utilized in the future to provide a complete view across varied abiotic stressors. The characterization of the key signaling SnRK2 kinases involved in abiotic stress tolerance is progressing using various methods, including traditional forward and reverse genetic assessments in numerous plant species, large-scale genome sequencing, proteomics data analysis, and genome engineering methods. We postulate that with a better grasp of SnRK2 kinase-mediated signaling pathways, we will successfully design and create climate-smart plants utilizing genetic improvement techniques to increase agricultural production requirements for the growing global population.

## References

[1] Hasan MM, Ali MA, Soliman MH, Alqarawi AA, Abd Allah EF, Fang X-W. Insights into 28-homobrassinolide (HBR)-mediated redox homeostasis, AsA–GSH cycle, and methylglyoxal detoxification in soybean under drought-induced oxidative stress. J Plant Inter. 2020a. 15:371–6. doi:10.1080/17429145.2020.1832267.

[2] Jahan MS, Wang Y, Shu S, Hasan MM, El-Yazied AA, Alabdallah NM, Hajjar D, Altaf MA, Sun J, Guo S. Melatonin pretreatment confers heat tolerance and repression of heat-induced senescence in tomato through the modulation of ABA. Front Plant Sci. 2021;12:650955. doi:10.3389/fpls.2021.650955. 33841479 PMC8027311

[3] Waseem M, Nie NF, Yao GQ, Hasan MM, Xiang Y, Fang XW. Dew absorption by leaf trichomes in *Caragana korshinskii*: an alternative water acquisition strategy for withstanding drought in arid environments. Physiol Plant. 2021;172(2):528–539. doi:10.1111/ppl.13334. 33452683

[4] Hasan MM, Skalicky M, Jahan MS, Hossain MN, Anwar Z, Nie ZF, Alabdallah NM, Brestic M, Hejnak V, Fang X-W. Spermine: its emerging role in regulating drought stress responses in plants. Cells. 2021a;10(2):261. doi:10.3390/cells10020261. 33525668 PMC7912026

[5] Khan A, Anwar Y, Hasan M, Iqbal A, Ali M, Alharby HF, Hakeem KR, Hasanuzzaman M. Attenuation of drought stress in Brassica seedlings with exogenous application of Ca^2+^ and H_2_O_2_. Plants. 2017;6(4):20. doi:10.3390/plants6020020. 28505096 PMC5489792

[6] Hasan MM, Alharby HF, Uddin MN, Ali MA, Anwar Y, Fang XW, Hakeem KR, Alzahrani Y, Hajar AS. Magnetized water confers drought stress tolerance in *Moringa* Biotype via modulation of growth, gas exchange, lipid peroxidation and antioxidant activity. Pol J Environ Stud. 2020b;29(2):1625–1636. doi:10.15244/pjoes/110347.

[7] Hasan MM, Alabdallah NM, Alharbi BM, Waseem M, Yao G, Liu X-D, El-Gawad HGA, El-Yazied AA, Ibrahim MFM, Jahan MS, et al. GABA: a key player in drought stress resistance in plants. Int J Mol Sci. 2021b;462:660–664. doi:10.3390/ijms221810136. PMC8471019 34576299

[8] Barajas-Lopez JD, Moreno JR, Gamez-Arjona FM, Pardo JM, Punkkinen M, Zhu JK, Quintero FJ, Fujii H. Upstream kinases of plant SnRKs are involved in salt stress tolerance. Plant J. 2018;93(1):107–118. doi:10.1111/tpj.13761. 29094495 PMC5814739

[9] Hasan MM, Gong L, Nie ZF, Feng X, Ahammed GJ, Fang X-W. ABA-induced stomatal movements in vascular plants during dehydration versus rehydration. Environ Exp Bot. 2021d;186:104436. doi:10.1016/j.envexpbot.2021.104436.

[10] Yoshida R, Umezawa T, Mizoguchi T, Takahashi S, Takahashi F, Shinozaki K. The regulatory domain of SRK2E/OST1/SnRK2.6 interacts with ABI1 and integrates abscisic acid (ABA) and osmotic stress signals controlling stomatal closure in *Arabidopsis*. J Biol Chem. 2006;281(8):5310–5318. doi:10.1074/jbc.M509820200. 16365038

[11] Umezawa T, Sugiyama N, Takahashi F, Anderson JC, Ishihama Y, Peck SC, Shinozaki K. Genetics and phosphoproteomics reveal a protein phosphorylation network in the abscisic acid signaling pathway in *Arabidopsis thaliana*. Sci Signal. 2013;6(270):rs8. doi:10.1126/scisignal.2003509. 23572148

[12] Wang P, Xue L, Batelli G, Lee S, Hou YJ, Van Oosten MJ, Zhang H, Tao WA, Zhu J-K. Quantitative phosphoproteomics identifies SnRK2 protein kinase substrates and reveals the effectors of abscisic acid action. Proc Natl Acad Sci U S A. 2013;110(27):11205–11210. doi:10.1073/pnas.1308974110. 23776212 PMC3703982

[13] Chen X, Ding Y, Yang Y, Song C, Wang B, Yang S, Guo Y, Gong Z. Protein kinases in plant responses to drought, salt, and cold stress. J Integr Plant Biol. 2021;63(1):53–78. doi:10.1111/jipb.13061. 33399265

[14] Dai M, Xue Q, Mccray T, Margavage K, Chen F, Lee JH, Nezames CD, Guo L, Terzaghi W, Wan J, et al. The PP6 phosphatase regulates ABI5 phosphorylation and abscisic acid signaling in *Arabidopsis*. Plant Cell. 2013;25(2):517–534. doi:10.1105/tpc.112.105767. 23404889 PMC3608775

[15] Shu K, Chen F, Zhou W, Luo X, Dai Y, Shuai H, Yang W. ABI4 regulates the floral transition independently of ABI5 and ABI3. Mol Biol Rep. 2018;45(6):2727–2731. doi:10.1007/s11033-018-4290-9. 30121823

[16] Hwang K, Susila H, Nasim Z, Jung JY, Ahn JH. Arabidopsis ABF3 and ABF4 transcription factors act with the NF-YC complex to regulate SOC1 expression and mediate drought-accelerated flowering. Mol Plant. 2019;12(4):489–505. doi:10.1016/j.molp.2019.01.002. 30639313

[17] Chong L, Xu R, Huang P, Guo P, Zhu M, Du H, Sun X, Ku L, Zhu J-K, Zhu Y. The tomato OST1-VOZ1 module regulates drought-mediated flowering. Plant Cell. 2022. doi:10.1093/plcell/koac026. PMC9048945 35099557

[18] Cutler SR, Rodriguez PL, Finkelstein RR, Abrams SR. Abscisic acid: emergence of a core signaling network. Ann Rev Plant Biol. 2009;61(1):651–679. doi:10.1146/annurevarplant-042809-112122. 20192755

[19] Fujii H, Zhu J-K. Arabidopsis mutant deficient in 3 abscisic acid-activated protein kinases reveals critical roles in growth, reproduction, and stress. Proc Natl Acad Sci USA. 2009;106(20):8380–8385. doi:10.1073/pnas.0903144106. 19420218 PMC2688869

[20] Wang P, Zhu JK, Lang Z. Nitric oxide suppresses the inhibitory effect of abscisic acid on seed germination by S-nitrosylation of SnRK2 proteins. Plant Signal Behav. 2015;10(6):e1031939. doi:10.1080/15592324.2015.1031939. 26024299 PMC4622540

[21] Garcia-Mata C, Gay R, Sokolovski S, Hills A, Lamattina L, Blatt MR. Nitric oxide regulates K+and Cl-channels in guard cells through a subset of abscisic acid-evoked signaling pathways. Proc Natl Acad Sci USA. 2003;100(19):11116–11121. PMID:12949257. doi:10.1073/pnas.1434381100. 12949257 PMC196936

[22] Rock CD, Sakata Y, Quatrano RS. Abiotic stress adaptation in plants. Pareek A, et al. eds. Netherlands: Springer; 2009. p. 33–73.

[23] Nakashima K, Fujita Y, Kanamori N, Katagiri T, Umezawa T, Kidokoro S, Maruyama K, Yoshida T, Ishiyama K, Kobayashi M, et al. Three Arabidopsis SnRK2 protein kinases, SRK2D/SnRK2.2, SRK2E/SnRK2.6/OST1 and SRK2I/SnRK2.3, involved in ABA signaling are essential for the control of seed development and dormancy. Plant Cell Physiol. 2009;50(7):1345–1363. doi:10.1093/pcp/pcp083. 19541597

[24] Fujita Y, Nakashima K, Yoshida T, Katagiri T, Kidokoro S, Kanamori N, Umezawa T, Fujita M, Maruyama K, Ishiyama K, et al. Three SnRK2 protein kinases are the main positive regulators of abscisic acid signaling in response to water stress in *Arabidopsis*. Plant Cell Physiol. 2009;50(12):2123–2132. doi:10.1093/pcp/pcp147. 19880399

[25] Umezawa T, Nakashima K, Miyakawa T, Kuromori T, Tanokura M, Shinozaki K, Yamaguchi-Shinozaki K. Molecular basis of the core regulatory network in ABA responses: sensing, signaling and transport. Plant Cell Physiol. 2010;51(11):1821–1839. doi:10.1093/pcp/pcq156. 20980270 PMC2978318

[26] Park SY, Fung P, Nishimura N, Jensen DR, Fujii H, Zhao Y, Lumba S, Santiago J, Rodrigues A, Chow TF, et al. Abscisic acid inhibits type 2C protein phosphatases via the PYR/PYL family of START proteins. Science. 2009;324(5930):1068–1071. doi:10.1126/science.1173041. 19407142 PMC2827199

[27] Kamiyama Y, Katagiri S, Umezawa T. Growth promotion or osmotic stress response: how SNF1-related protein kinase 2 (SnRK2) kinases are activated and manage intracellular signaling in plants. Plants. 2021;10(7):1443. doi:10.3390/plants10071443. 34371646 PMC8309267

[28] Mizoguchi M, Umezawa T, Nakashima K, Kidokoro S, Takasaki H, Fujita Y, Yamaguchi-Shinozaki K, Shinozaki K. Two closely related subclass II SnRK2 protein kinases cooperatively regulate drought-inducible gene expression. Plant Cell Physiol. 2010;51(5):842–847. doi:10.1093/pcp/pcq041. 20375108

[29] Li J, Wang XQ, Watson MB, Assmann SM. Regulation of abscisic acid-induced stomatal closure and anion channels by guard cell AAPK kinase. Science. 2000;287(5451):300–303. doi:10.1126/science.287.5451.300. 10634783

[30] Chen K, Li GJ, Bressan RA, Song CP, Zhu JK, Zhao Y. Abscisic acid dynamics, signaling, and functions in plants. J Integr Plant Biol. 2020;62(1):25–54. doi:10.1111/jipb.12899. 31850654

[31] Hasan MM, Rahman MA, Skalicky M, Alabdallah NM, Waseem M, Jahan MS, Ahammed GJ, El-Mogy MM, El-Yazied AA, Ibrahim MFM, et al. Ozone induced stomatal regulations, MAPK and phytohormone signaling in plants. Int J Mol Sci. 2021c;22(12):6304. doi:10.3390/ijms22126304. 34208343 PMC8231235

[32] Vlad F, Rubio S, Rodrigues A, Sirichandra C, Belin C, Robert N, Leung J, Rodriguez PL, Laurière C, Merlot S. Protein phosphatases 2C regulate the activation of the Snf1-related kinase OST1 by abscisic acid in arabidopsis. Plant Cell. 2009;21(10):3170–3184. doi:10.1105/tpc.109.069179. 19855047 PMC2782292

[33] Fujii H, Chinnusamy V, Rodrigues A, Rubio S, Antoni R, Park SY, Cutler SR, Sheen J, Rodriguez PL, Zhu J-K. In vitro reconstitution of an abscisic acid signalling pathway. Nature. 2009;462(7273):660–664. doi:10.1038/nature08599. 19924127 PMC2803041

[34] Rensing SA, Lang D, Zimmer AD, Terry A, Salamov A, Shapiro H, Nishiyama T, Perroud PF, Lindquist EA, Kamisugi Y, et al. The Physcomitrella genome reveals evolutionary insights into the conquest of land by plants. Science. 2008;4319(5859):64–69. doi:10.1126/science.1150646. Epub 2007 Dec 13. PMID: 18079367. 18079367

[35] Chater C, Kamisugi Y, Movahedi M, Fleming A, Cuming AC, Gray JE, Beerling DJ. Regulatory mechanism controlling stomatal behavior conserved across 400 million years of land plant evolution. Curr Biol. 2011;21(12):1025–1029. doi:10.1016/j.cub.2011.04.032. 21658944

[36] Shinozawa A, Otake R, Takezawa D, Umezawa T, Komatsu K, Tanaka K, Amagai A, Ishikawa S, Hara Y, Kamisugi Y, et al. SnRK2 protein kinases represent an ancient system in plants for adaptation to a terrestrial environment. Commun Biol. 2019;2(1):30. doi:10.1038/s42003-019-0281-1. 30675528 PMC6340887

[37] Kulik A, Wawer I, Krzywinska E, Bucholc M, Dobrowolska G. SnRK2 protein kinases-key regulators of plant response to abiotic stresses. OMICS. 2011;15(12):859–872. doi:10.1089/omi.2011.0091. 22136638 PMC3241737

[38] Saruhashi M, Kumar Ghosh T, Arai K, Ishizaki Y, Hagiwara K, Komatsu K, Shiwa Y, Izumikawa K, Yoshikawa H, Umezawa T, et al. Plant Raf-like kinase integrates abscisic acid and hyperosmotic stress signaling upstream of SNF1-related proteinkinase2. Proc Natl Acad Sci USA. 2015;112(46):201511238–E6396. doi:10.1073/pnas.1511238112. PMC4655548 26540727

[39] Hori K, Maruyama F, Fujisawa T, Togashi T, Yamamoto N, Seo M, Sato S, Yamada T, Mori H, Tajima N, et al. Klebsormidium flaccidum genome reveals primary factors for plant terrestrial adaptation. Nat Commun. 2014;5(1):3978. doi:10.1038/ncomms4978. 24865297 PMC4052687

[40] Yoshida R, Hobo T, Ichimura K, Mizoguchi T, Takahashi F, Aronso J, Ecker JR, Shinozaki K. ABA-activated SnRK2 protein kinase is required for dehydration stress signaling in Arabidopsis. Plant Cell Physiol. 2002;43(12):1473–1483. doi:10.1093/pcp/pcf188. 12514244

[41] Mustilli AC, Merlot S, Vavasseur A, Frenzi F, Giraudat J. Arabidopsis OST1 protein kinase mediates the regulation of stomatal aperture by abscisic acid and acts upstream of reactive oxygen species production. Plant Cell. 2002;14(12):3089–3099. doi:10.1105/tpc.007906. 12468729 PMC151204

[42] Kwak JM, Mori IC, Pei ZM, Leonhardt N, Torres MA, Dangl JL, Bloom RE, Bodde S, Jones JDG, Schroeder JI, et al. NADPH oxidase AtrbohD and AtrbohF genes function in ROS-dependent ABA signaling in Arabidopsis. EMBO J. 2003;22(11):2623–2633. doi:10.1093/emboj/cdg277. 12773379 PMC156772

[43] Gong L, Liu XD, Zeng YY, Tian XQ, Li YL, Turner NC, Fang XW et al. Stomatal morphology and physiology explain varied sensitivity to abscisic acid across vascular plant lineages. Plant Physiol. 2021;186(1):782–797. doi:10.1093/plphys/kiab090. 33620497 PMC8154066

[44] Sirichandra C, Gu D, Hu HC, Davanture M, Lee S, Djaoui M, Valot B, Zivy M, Leung J, Merlot S, et al. Phosphorylation of the Arabidopsis AtrbohF NADPH oxidase by OST1 protein kinase. FEBS Lett. 2009;583(18):2982–2986. doi:10.1016/j.febslet.2009.08.033. 19716822

[45] Negi J, Matsuda O, Nagasawa T, Oba Y, Takahashi H, Kawai-Yamada M, Uchimiya H, Hashimoto M, Iba K. CO_2_ regulator SLAC1 and its homologues are essential for anion homeostasis in plant cells. Nature. 2008;452(7186):483–486. doi:10.1038/nature06720. 18305482

[46] Vahisalu T, Kollist H, Wang YF, Nishimura N, Chan WY, Valerio G, Lamminmäki A, Brosché M, Moldau H, Desikan R, et al. SLAC1 is required for plant guard cell S-type anion channel function in stomatal signalling. Nature. 2008;452(7186):487–491. doi:10.1038/nature06608. 18305484 PMC2858982

[47] Geiger D, Scherzer S, Mumm P, Stange A, Marten I, Bauer H, Ache P, Matschi S, Liese A, Al-Rasheid KAS, et al. Activity of guard cell anion channel SLAC1 is controlled by drought-stress signaling kinase-phosphatase pair. Proc Natl Acad Sci USA. 2009;106(50):21425–21430. doi:10.1073/pnas.0912021106. 19955405 PMC2795561

[48] Pilot G, Lacombe B, Gaymard F, Cherel I, Boucherez J, Thibaud JB, Sentenac H. Guard cell inward K+ channel activity in Arabidopsis involves expression of the twin channel subunits KAT1 and KAT2. J Biol Chem. 2001;276(5):3215–3221. doi:10.1074/jbc.M007303200. 11042178

[49] Zhang Q, Shao Q, Guo Y, Li N, Li Y, Su J, Xu R, Zhang Z, Xiao L, Feng Y. Characterization of three calcium-dependent protein kinases of cryptosporidium parvum. Front Microbiol. 2021;11:3467. doi:10.3389/fmicb.2020.622203. PMC7835281 33510735

[50] Vahisalu T, Puzõrjova I, Brosché M, Valk E, Lepiku M, Moldau H, Pechter P, Wang YS, Lindgren O, Salojärvi J, et al. Ozone-triggered rapid stomatal response involves the production of reactive oxygen species, and is controlled by SLAC1 and OST1. Plant J. 2010;62(3):442–453. doi:10.1111/j.1365-313X.2010.04159.x. 20128877

[51] Xue S, Hu H, Ries A, Merilo E, Kollist H, Schroeder JI. Central functions of bicarbonate in S-type anion channel activation and OST1 protein kinase in CO2 signal transduction in guard cell. EMBO J. 2011;30(8):1645–1658. doi:10.1038/emboj.2011.68. 21423149 PMC3102275

[52] Wang P, Zhao Y, Li Z, Hsu CC, Liu X, Fu L, Hou YJ, Du Y, Xie S, Zhang C, et al. Reciprocal regulation of the TOR kinase and ABA receptor balances plant growth and stress response. Mol Cell. 2018;69(1):100–112. doi:10.1016/j.molcel.2017.12.002. 29290610 PMC5772982

[53] Belda-Palazón B, Adamo M, Valerio C, Ferreira LJ, Confraria A, Reis-Barata D, Rodrigues A, Meyer C, Rodriguez PL, Baena-Gonzalez E. A dual function of SnRK2 kinases in the regulation of SnRK1 and plant growth. Nat Plants. 2020;6(11):1345–1353. doi:10.1038/s41477-020-00778-w. 33077877

[54] Yoshida T, Obata T, Feil R, Lunn JE, Fujita Y, Yamaguchi-Shinozaki K, Fernie AR. The role of abscisic acid signaling in maintaining the metabolic balance required for Arabidopsis growth under nonstress conditions. Plant Cell. 2019;31(1):84–105. doi:10.1105/tpc.18.00766. 30606780 PMC6391705

[55] Zheng Z, Xu X, Crosley RA, Greenwalt SA, Sun Y, Blakeslee B, Wang L, Ni W, Sopko MS, Yao C, et al. The protein kinase SnRK2.6 mediates the regulation of sucrose metabolism and plant growth in Arabidopsis. Plant Physiol. 2010;153(1):99–113. doi:10.1104/pp.109.150789. 20200070 PMC2862418

[56] Rodrigues A, Adamo M, Crozet P, Margalha L, Confraria A, Martinho C, Elias A, Rabissi A, Lumbreras V, González-Guzmán M, et al. ABI1 and PP2CA phosphatases are negative regulators of Snf1-related protein kinase1 signaling in Arabidopsis. Plant Cell. 2013;25(10):3871–3884. doi:10.1105/tpc.113.114066. 24179127 PMC3877788

